# Visceral Adiposity, Rather than Reduced Appendicular Lean Mass, Characterizes Elderly Hip Fracture Patients with Type 2 Diabetes: A Cross-Sectional DXA Analysis

**DOI:** 10.3390/jcm15062284

**Published:** 2026-03-17

**Authors:** Hyuna Kang, Minkyu Choi, Youngkyun Roh, Yonghyun Yoon, Jihyo Hwang

**Affiliations:** 1 Department of Family Medicine, Gangnam Sacred Heart Hospital, College of Medicine, Hallym University, Seoul 07441, Republic of Korea; 2 Department of Orthopaedic Surgery, Gangnam Sacred Heart Hospital, College of Medicine, Hallym University, Seoul 07441, Republic of Korea; 3 Incheon Terminal Orthopedic Surgery Clinic, Inha-ro 489beon-gil, Namdong-gu, Incheon 21574, Republic of Korea; 4 International Academy of Regenerative Medicine, Inha-ro 489beon-gil, Namdong-gu, Incheon 21574, Republic of Korea; 5 International Association of Musculoskeletal Medicine, Kowloon, Hong Kong, China; 6 MSKUS, 1035 E. Vista Way #128, Vista, CA 92084, USA

**Keywords:** type 2 diabetes mellitus, hip fracture, body composition, visceral fat, sarcopenia, abdominal muscle, DXA

## Abstract

**Background/Objectives:** While sarcopenia has been implicated, we hypothesize that a distinct body composition phenotype, characterized by elevated visceral adiposity and reduced abdominal muscle mass, plays a more critical role in T2DM-related fracture pathogenesis. **Methods:** In a cross-sectional study of 99 female patients aged ≥65 years who underwent surgery for hip fracture, we compared body composition parameters derived from DXA scans between those with (*n* = 40) and without (*n* = 59) T2DM. Key measures included appendicular lean mass index (ALMI), visceral adipose tissue (VAT) mass, android-to-gynoid (A/G) fat ratio, and a derived measure of relative core lean mass (RCLM). **Results:** There were no significant differences in ALMI between T2DM and non-DM groups. In contrast, T2DM showed significantly higher central adiposity—A/G ratio (1.13 ± 0.15 vs. 1.05 ± 0.17; *p* = 0.0298) and TL fat ratio (1.31 ± 0.22 vs. 1.19 ± 0.23; *p* = 0.0071)—with VAT estimate numerically higher. **Conclusions:** In older hip-fracture patients, T2DM was characterized not by appendicular sarcopenia but by central adiposity without significant differences in LMI or RCLM—a phenotype that may contribute to fracture risk through bone-quality and fall-related pathways independent of ALMI.

## 1. Introduction

Hip fracture remains one of the most devastating events in older adults, leading to excess mortality, prolonged disability, and substantial healthcare costs [1,2,3]. Type 2 diabetes mellitus (T2DM) is consistently associated with higher fragility-fracture risk [4,5,6], especially at the hip—despite the oft-noted “diabetic bone paradox,” whereby bone mineral density (BMD) is normal or even higher in T2DM compared with non-diabetes [7,8,9]. Multiple large-scale epidemiologic studies have demonstrated that individuals with T2DM exhibit increased fracture risk despite preserved or elevated BMD [7,9,10,11]. Emerging evidence suggests that impaired bone material properties, cortical porosity, and accumulation of advanced glycation end-products contribute to skeletal fragility independent of areal BMD. These observations indicate that fracture susceptibility in diabetes extends beyond mineral density and may involve bone-quality deficits and extra-skeletal contributors such as altered body composition and fall propensity [10,12,13]. Hip fractures predominantly affect postmenopausal women, who also exhibit a higher prevalence of osteoporosis [14,15].

Sarcopenia has long been considered a prime suspect linking diabetes to fractures [16,17]. However, contemporary consensus definitions (e.g., EWGSOP2) emphasize that sarcopenia is characterized not only by reduced muscle quantity but also by impaired muscle strength and/or physical performance. In many epidemiologic studies, appendicular lean mass index (ALMI) derived from DXA is used as a surrogate marker of muscle quantity, although it does not capture muscle distribution, intramuscular fat infiltration, or functional capacity [18,19].

In parallel, type 2 diabetes mellitus (T2DM) is characterized by a distinct remodeling of body composition, with preferential accumulation of central adiposity—particularly visceral adipose tissue (VAT)—and associated metabolic inflammation [20,21,22]. VAT functions as an active endocrine organ that secretes pro-inflammatory cytokines and adipokines, exacerbates insulin resistance, and may adversely influence both bone and muscle biology [10,20,23]. In addition, trunk and abdominal musculature contribute to postural stability and balance control [24,25]. Therefore, alterations in fat distribution and lean-mass patterning—rather than isolated reductions in appendicular muscle quantity—may influence fall propensity and fracture vulnerability in diabetes, even when ALMI appears preserved.

Based on this framework, we asked whether, in older hip-fracture patients, T2DM is necessarily associated with reduced appendicular lean mass or whether central adiposity represents a more salient compositional feature. To address this question, we analyzed DXA-derived body composition parameters in 99 hip-fracture patients (T2DM *n* = 40; non-DM *n* = 59). In addition to standard indices (ALMI, LMI, VAT estimate, A/G ratio, trunk-to-limb fat ratio), we explored a proportional distribution metric—relative central lean mass (RCLM = 100 × [LMI − ALMI]/LMI)—as an exploratory indicator of lean distribution rather than a validated measure of trunk skeletal muscle.

## 2. Materials and Methods

### 2.1. Study Design and Population

This retrospective cross-sectional study was conducted at a single tertiary care center. The study protocol was reviewed and approved by the Institutional Review Board (IRB) of Hallym University Kangnam Sacred Heart Hospital, which waived the requirement for informed consent because of the retrospective nature of the analysis.

We initially screened the electronic medical records of 235 consecutive female patients aged ≥65 years who underwent surgical treatment for hip fracture at our institution between August 2020 and July 2025. All eligible patients had undergone dual-energy X-ray absorptiometry (DXA) as part of routine post-fracture evaluation for bone health and body composition.

Sarcopenia was not formally diagnosed because functional assessments such as muscle strength or physical performance tests were not available in this retrospective dataset.

### 2.2. Participant Selection and Exclusion Criteria

**Figure 1:**
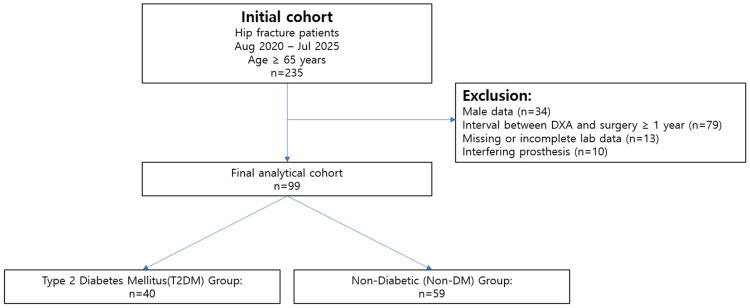
Flowchart of Patient Selection.

The primary aim of the selection process was to identify a cohort with reliable and contemporaneous DXA-based body composition data. The detailed patient selection process is outlined in Figure 1. From the initial cohort of 235 patients, 136 were excluded based on the following pre-defined criteria:

1. Male patients were excluded to focus on a female cohort (*n* = 34).
2. A time interval of over one year between the date of surgery and the date of the DXA scan, to ensure body composition data reflected the peri-fracture state (*n* = 79).
3. Missing or incomplete data in key variables of interest (*n* = 13).
4. The presence of extensive hip, spine, or knee prostheses that could potentially interfere with accurate regional BMD or body composition measurement (*n* = 10).

After applying these exclusion criteria, the final study population comprised 99 patients who were included in the statistical analysis. This cohort was subsequently divided into two groups based on diabetes status: the T2DM group (*n* = 40) and the non-diabetic (non-DM) group (*n* = 59) (Figure 1).

### 2.3. DXA Measurements

Whole-body DXA scans were performed using a Hologic Horizon W densitometer (Serial No. 200630, Hologic Inc., Marlborough, MA, USA) with software version 13.6.0.4. The scans were used for body composition analysis in the present study. Body-composition outputs included fat mass index (FMI), appendicular lean mass index (ALMI), lean mass index (LMI), and fat distribution indices, including the android-to-gynoid (A/G) fat ratio and trunk-to-limb (TL) fat mass ratio.

Visceral adipose tissue (VAT) estimates were obtained using the manufacturer’s CoreScan algorithm embedded in the Hologic system [26]. The software provides VAT area and volume estimates derived from proprietary modeling of abdominal fat distribution. Although VAT area has been validated against CT and MRI in previous studies, the volumetric outputs are algorithm-derived and should not be interpreted as directly equivalent to CT-derived visceral fat measurements [20].

To assess lean distribution, we defined relative central lean mass (RCLM) as 100 × (LMI − ALMI)/LMI, representing the proportion of non-appendicular lean mass within total lean mass derived from DXA indices. RCLM is an exploratory distribution metric and not a validated surrogate for trunk skeletal muscle mass.

All scans were acquired under routine clinical densitometry protocols at our institution, including manufacturer-recommended calibration and daily quality-control procedures. According to the institutional densitometry protocol, the short-term coefficient of variation (CV) for whole-body DXA measurements is approximately 1.0% for total body fat and 0.8–1.2% for lean mass measurements.

All patient and site identifiers (including dates and device serial information) were removed or masked; acquisition parameters and device details are provided in the Methods.

Abbreviations (DXA body composition): FMI, fat mass index; LMI, lean mass index; ALMI, appendicular lean mass index; A/G, android-to-gynoid fat ratio; TL, trunk-to-limb fat ratio; VAT, visceral adipose tissue; RCLM, relative central lean mass.

**Figure 2:**
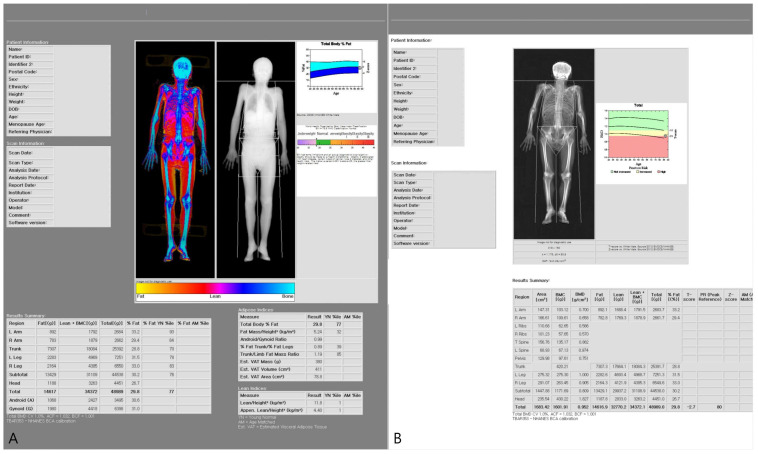
Representative whole-body DXA body composition output used in the study. (**A**,**B**) Body composition report displaying color-mapped lean and fat distribution and derived adiposity indices, including the android-to-gynoid (A/G) fat ratio, trunk-to-limb (TL) fat mass ratio, and estimated visceral adipose tissue (VAT) derived from the CoreScan algorithm (area, mass, and volume estimates).

Representative DXA body composition outputs are shown in Figure 2.

### 2.4. Statistical Analysis

Continuous variables are presented as mean ± standard deviation and were compared between groups using Welch’s *t*-test. Categorical variables were analyzed using the chi-square test or Fisher’s exact test, as appropriate. Effect sizes are reported as standardized mean differences (Hedges’ g).

**Figure 3:**
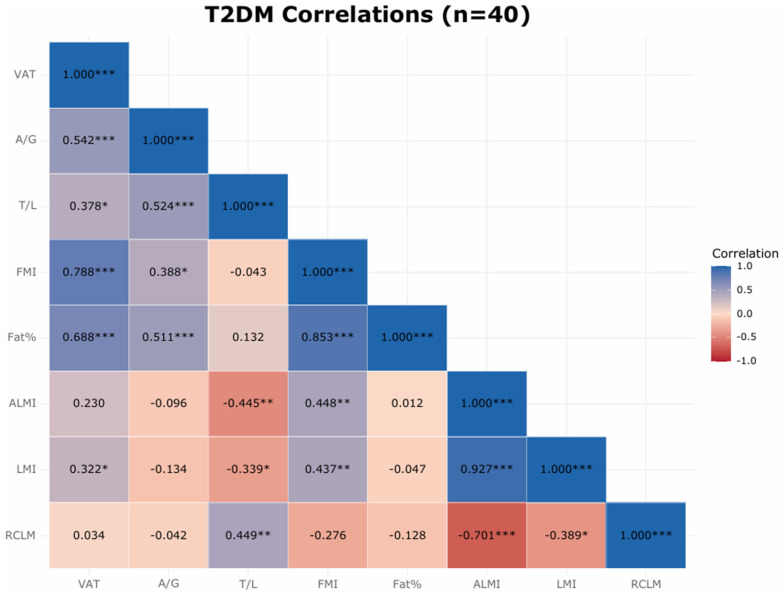
Within-diabetes correlation heatmap (T2DM subgroup, *n* = 40). Cells display Pearson’s correlation coefficients (r). Significance levels are indicated by asterisks (* *p* < 0.05, ** *p* < 0.01, *** *p* < 0.001). Variables included visceral adipose tissue (VAT) estimate, android-to-gynoid (A/G) fat ratio, trunk-to-limb (TL) fat ratio, fat mass index (FMI), total body fat percentage, appendicular lean mass index (ALMI), lean mass index (LMI), relative central lean mass (RCLM), and HbA1c.

Within the T2DM subgroup, Pearson correlation coefficients were calculated to assess relationships among glycemic control (HbA1c), adiposity indices (VAT estimate, A/G ratio, trunk-to-limb fat ratio), and lean mass measures (ALMI, LMI, RCLM). Pairwise associations were visualized using scatterplots with ordinary least-squares regression lines (Supplementary Figures S1–S5), and correlation matrices were visualized using a heatmap (Figure 3). Pearson’s r and corresponding two-sided *p*-values are reported in Supplementary Table S1 and in figure captions.

Statistical significance was defined as a two-sided *p*-value < 0.05. Sensitivity analyses were conducted using multivariable linear regression models adjusting for age and BMI to assess the robustness of key associations. Because the cohort size was determined by consecutive clinical availability, no a priori sample size calculation was performed. Post hoc achieved-power analyses (two-sided α = 0.05) were conducted for primary between-group comparisons, with results provided in Supplementary Table S2. All analyses were performed in Python (version 3.8).

## 3. Results

### 3.1. Participant Characteristics

Ninety-nine patients were analyzed (T2DM, *n* = 40; non-DM, *n* = 59). As shown in Table 1, groups were comparable in age (79.62 ± 8.82 vs. 78.69 ± 9.86 years; *p* = 0.6247) and BMI (23.14 ± 3.84 vs. 21.80 ± 4.36 kg/m^2^; *p* = 0.1100). Body weight was higher in T2DM (56.09 ± 10.13 vs. 51.52 ± 10.85 kg; *p* = 0.0355). HbA1c was significantly higher in the T2DM group (7.05 ± 1.27% vs. 5.68 ± 0.44%; *p* < 0.001). The trunk-to-limb fat ratio was also significantly higher in T2DM patients (1.31 ± 0.22 vs. 1.19 ± 0.23; *p* = 0.007).

### 3.2. Body Composition Between Groups

Central adiposity metrics were higher in the T2DM group. The android-to-gynoid (A/G) fat ratio was greater in T2DM than in non-DM (1.13 ± 0.15 vs. 1.05 ± 0.17; *p* = 0.030). Similarly, the trunk-to-limb (TL) fat mass ratio was higher in T2DM (1.31 ± 0.22 vs. 1.19 ± 0.23; *p* = 0.007). Estimated VAT was numerically higher in T2DM (658.84 ± 308.81 vs. 555.37 ± 259.79; *p* = 0.086).

In contrast, lean-mass indices did not significantly differ between groups: ALMI (5.11 ± 0.94 vs. 5.07 ± 0.85; *p* = 0.821), LMI (13.28 ± 1.81 vs. 12.95 ± 1.63; *p* = 0.367), and RCLM (61.63 ± 2.82 vs. 60.92 ± 3.21; *p* = 0.254). Overall, the T2DM group demonstrated greater central adiposity without significant differences in appendicular or total lean indices. RCLM was interpreted as an exploratory DXA-derived lean distribution metric.

### 3.3. Within-Diabetes Correlation Analysis (Updated)

In the T2DM subgroup (*n* = 40), correlation analyses revealed two coherent patterns (Figure 3). First, indices of visceral/central adiposity were positively correlated: VAT estimate correlated positively with overall adiposity (FMI, fat %) and with fat distribution measures (A/G ratio and trunk-to-limb fat ratio), and A/G ratio correlated positively with trunk-to-limb fat ratio. Second, lean-mass patterning showed a reciprocal relationship with central fat distribution: ALMI was positively associated with FMI but inversely associated with trunk-to-limb fat ratio, suggesting relatively greater central fat distribution when appendicular lean prominence is lower. As expected, LMI and ALMI were strongly correlated. Statistical significance was evaluated using two-sided *p*-values.

Glycemia showed no statistically significant cross-sectional associations with VAT estimate, A/G ratio, or trunk-to-limb fat ratio, indicating that these composition patterns were largely independent of near-term HbA1c.

These findings remained directionally consistent in sensitivity analyses adjusting for age and BMI. Collectively, the heatmap supports a central adiposity cluster (VAT estimate, A/G ratio, trunk-to-limb fat ratio, and overall adiposity) that appears largely decoupled from HbA1c, alongside a lean-distribution signal in which ALMI varies inversely with central fat distribution. These observations suggest that fat and lean distribution patterns may provide complementary information beyond HbA1c for characterizing the body-composition profile within diabetes (Figure 3; Supplementary Figures S1–S5).

## 4. Discussion

### 4.1. Principal Findings

In this cohort of older adults with hip fracture, T2DM was characterized primarily by greater central (abdominal) adiposity without a significant reduction in appendicular lean mass. ALMI did not significantly differ between groups, whereas A/G ratio and trunk-to-limb fat ratio were significantly higher in T2DM, and VAT estimates were numerically higher. LMI and RCLM did not significantly differ between groups. Given the modest sample size, small differences in lean distribution cannot be excluded; however, no directional inference is made. Because standardized functional measures were not available, we did not diagnose sarcopenia according to consensus criteria. Accordingly, our findings should be interpreted as between-group differences in DXA-derived lean mass parameters rather than the presence or absence of sarcopenia.

Within the T2DM subgroup, correlation analyses demonstrated clustering among central adiposity indices (VAT estimate, FMI, fat percentage, A/G ratio, and trunk-to-limb fat ratio), whereas HbA1c showed no meaningful cross-sectional associations with body composition measures. ALMI demonstrated positive association with overall adiposity (FMI) and inverse association with trunk-to-limb fat ratio, suggesting a redistribution pattern rather than isolated appendicular muscle loss.

This study focused on body composition patterns derived from DXA rather than bone mineral density measurements. The findings suggest that fracture vulnerability in patients with T2DM may relate more closely to fat distribution patterns and potential fall-related mechanisms than to reductions in appendicular lean mass alone.

### 4.2. Interpretation in the Context of Prior Literature and Mechanisms

#### 4.2.1. Context Within the Existing Literature

Community-based studies have reported a higher prevalence of sarcopenia among individuals with T2DM, typically defined using appendicular lean mass in combination with functional measures such as grip strength or gait speed [7,9]. At the same time, large epidemiologic cohorts have consistently demonstrated increased fracture risk in diabetes despite preserved or even elevated areal BMD [10]. This “diabetic bone paradox” has been further contextualized in mechanistic reviews highlighting altered bone material properties, cortical porosity, and advanced glycation end-product accumulation as contributors to skeletal fragility independent of mineral density [10].

However, these prior studies largely examined ambulatory or community-dwelling populations rather than individuals who had already sustained hip fracture. In contrast, our cohort consists of older adults post–hip fracture, a clinical state characterized by frailty, reduced mobility, and acute catabolic stress. These contextual differences may partially explain why appendicular lean mass did not significantly differ between groups in our study, despite findings of higher sarcopenia prevalence in community-based diabetes cohorts [17,27]. Accordingly, our results should be interpreted within the specific clinical context of a hip-fracture population.

#### 4.2.2. Central Adiposity as a Fracture Co-Driver

VAT estimate was strongly correlated with overall adiposity (FMI, Fat %) and with more adverse fat distribution (higher A/G ratio, higher TL fat ratio), consistent with the central adiposity signature in T2DM (Figure 3). Mechanistically, VAT-derived cytokines (e.g., IL-6, TNF-α) and advanced glycation end-products (AGEs) can degrade bone quality by promoting remodeling imbalance and collagen-cross-link alterations, while concomitantly aggravating insulin resistance—an axis coherent with diabetic bone fragility despite preserved or higher BMD at some sites [10,22,28]. Importantly, this mechanistic framework aligns with prior reports describing increased fracture risk in patients with T2DM despite preserved or even normal bone mineral density, suggesting that skeletal fragility in diabetes may reflect impaired bone quality rather than reduced mineral density alone.

#### 4.2.3. Core Musculature and Falls

Although between-group differences in lean indices did not reach statistical significance, appendicular and total lean mass appeared preserved in T2DM (Table 1), indicating preserved appendicular and overall lean mass rather than reduced appendicular lean mass. Even so, under central adiposity (greater mass inertia and altered biomechanics), the fall threshold may be lowered despite adequate limb muscle quantity [29]. Increased abdominal mass may shift the center of gravity anteriorly and increase trunk inertia during perturbation, potentially impairing balance recovery responses in older adults.

Consistent with this, Figure 3 shows an inverse relationship between ALMI and TL fat ratio and a tight clustering of VAT estimate, A/G ratio, and TL fat ratio, supporting a reciprocal distribution in which more centrally distributed fat accompanies relatively less peripheral-lean prominence in the composition profile [30,31,32]. In clinical terms, these patterns argue that where lean and fat are located—especially truncal support under central loading—matters more for falls than a small, non-significant difference in lean mass magnitude.

#### 4.2.4. Glycemia vs. Composition

Although HbA1c was higher in T2DM than in non-DM (Table 1), within the T2DM subgroup HbA1c showed no meaningful cross-sectional associations with VAT estimate, A/G ratio, or TL fat ratio on the heatmap (Figure 3). This decoupling indicates that the composition phenotype reflects a more persistent metabolic–mechanical state rather than near-term glycemia.

#### 4.2.5. Bone Density Within Diabetes

Within the T2DM subgroup, indices of central adiposity—including VAT estimate, A/G ratio, trunk-to-limb fat ratio, FMI, and total body fat percentage—showed strong positive correlations with each other on the heatmap (Figure 3). In contrast, lean mass indices such as ALMI and LMI demonstrated different correlation patterns, suggesting that lean mass distribution represents a distinct component of body composition.

Prior studies have reported that individuals with T2DM may exhibit increased fracture risk despite preserved or even normal bone mineral density, a phenomenon often referred to as the “diabetic bone paradox” [9,10,12]. These observations suggest that fracture susceptibility in diabetes may involve factors beyond areal BMD, including alterations in bone quality and fall-related biomechanics.

### 4.3. Interpretation and Mechanistic Considerations

In older adults with T2DM, assessment of body composition parameters such as A/G ratio, trunk-to-limb fat ratio, and VAT estimates may provide complementary information beyond ALMI alone. These indices are readily available from routine DXA reports and may help characterize the central adiposity pattern observed in this cohort.

The coexistence of central adiposity with preserved appendicular lean mass suggests that trunk stability and balance may represent relevant components of post-fracture rehabilitation. In patients with limited early mobility, low-intensity trunk activation and balance-focused exercises may be considered within standard rehabilitation protocols, with progression according to clinical status.

As HbA1c showed limited cross-sectional association with adiposity indices in this study, glycemic control alone may not fully reflect body-composition patterns. Interventions targeting visceral adiposity and fat distribution warrant prospective investigation, and future studies should evaluate whether combined metabolic and rehabilitation approaches influence secondary fracture risk.

Communication with patients may benefit from emphasizing body composition patterns rather than limb muscle quantity alone. Where feasible, follow-up DXA-derived composition metrics (e.g., VAT estimate, TL fat ratio, RCLM) may assist longitudinal monitoring, although their clinical utility requires further validation.

### 4.4. Context Within the Existing Literature

Whereas much of the diabetes–fracture literature emphasizes limb muscle loss or absolute BMD, our analysis foregrounds distribution: fat centralized in the abdomen and lean proportion shifted away from truncal support. By situating ALMI next to RCLM, A/G ratio, TL fat ratio, and VAT estimate, we show that the diabetic signature in hip-fracture patients is characterized primarily by metabolic and mechanical distribution patterns rather than strictly peripheral [17,31,33]. This perspective offers a plausible explanation for hip-fracture vulnerability in T2DM despite preserved ALMI and often higher BMD, consistent with a bone-quality and fall-mechanics pathway.

Perhaps a notable difference with prevailing reports is the preservation of ALMI in our T2DM cohort. Prior population-based studies—including the Korean Sarcopenic Obesity Study (KSOS)—have linked T2DM to a higher prevalence of sarcopenia defined by low ALMI [17,27,34,35]. Our findings do not negate those data but may reflect differences in study population and clinical context of older hip-fracture patients, in whom the phenotype appears central rather than appendicular. Several factors may account for this discrepancy: (i) our cohort represents a frailer, post-fracture population in which central adiposity and truncal support—rather than limb mass magnitude—are more proximate to falls; (ii) the catabolic/immobilization burden following fracture may partially homogenize appendicular lean across groups, obscuring pre-existing differences; and (iii) the coexistence of preserved ALMI with modestly lower LMI and a borderline lower RCLM underscores a qualitative remodeling of composition—where lean is located—rather than a purely quantitative loss.

Finally, our within-diabetes correlations (Figure 3) illustrate that most prior studies lack: VAT estimate clustered strongly with FMI/Fat % and A/G/TL (the central adiposity cluster), whereas HbA1c showed no meaningful cross-sectional associations with these indices. Together, these observations argue that risk stratification for secondary fracture prevention in T2DM may extend beyond quantifying appendicular muscle and explicitly interrogate the fat distribution patterns—a phenotype that is already accessible on routine DXA through VAT estimate, A/G ratio, TL fat ratio, and RCLM.

### 4.5. Clinical Implications

In the acute post-fracture period, many older adults are unable to participate in moderate-to-vigorous physical exercise due to pain, surgical recovery, or mobility limitations. Therefore, early management strategies should emphasize safe mobilization within orthopedic precautions and adequate nutritional support to prevent further lean mass loss. Ensuring sufficient energy and protein intake (typically 1.0–1.2 g/kg/day, individualized according to renal function and clinical status) may help mitigate catabolic muscle loss during immobilization. These recommendations are consistent with established geriatric and fracture-recovery guidelines and are not specific therapeutic claims derived from the present study.

As mobility improves, progressive weight-bearing, balance training, and trunk-stabilization exercises may be introduced according to rehabilitation protocols. Given the observed association between central adiposity indices and diabetes status in this cohort, structured aerobic conditioning during recovery may warrant investigation in future prospective studies.

Finally, follow-up assessment using repeat DXA body-composition analysis may help characterize changes in fat distribution and lean mass during recovery, although the clinical utility of such monitoring requires further validation.

Accordingly, glycemic control reflected by HbA1c alone may not fully capture central adiposity or lean distribution [20,36]; VAT-lowering or fat-redistributive approaches, paired with targeted core strengthening, are more likely to act on the pathways most relevant to fracture risk.

### 4.6. Strengths and Limitations

Strengths of this study include minimizing temporal misclassification by obtaining DXA-based body composition measurements during the same admission, analyzing fat/lean distribution indices (A/G ratio, trunk-to-limb fat ratio, VAT estimate, and RCLM) alongside ALMI, and conducting predefined within-diabetes correlation analyses (Figure 3). Key limitations include the cross-sectional design, absence of functional and bone-quality assessments (e.g., grip strength/gait speed, TBS/HR-pQCT), and unavailability of intramuscular fat and direct DXA trunk lean measures. In addition, incomplete diabetes-related clinical data (medication subclasses, diabetes duration, glycemic variability), fall history, and dietary protein intake precluded EWGSOP2-based sarcopenia classification and limited attribution of findings to disease versus treatment effects. VAT was derived from a DXA-based manufacturer algorithm rather than CT or MRI, and volumetric estimates should not be interpreted as directly equivalent to cross-sectional imaging–derived visceral fat measurements. Finally, this single-center cohort had a modest sample size and limited power to detect small effects (achieved power: 0.155 for LMI and 0.202 for RCLM; Supplementary Table S2); therefore, non-significant differences should not be interpreted as evidence of equivalence. Findings remained directionally consistent after sensitivity analyses adjusting for age and BMI, and external validation in larger prospective cohorts is warranted.

### 4.7. Future Directions

Prospective studies should integrate bone quality endpoints (TBS, HR-pQCT) with VAT estimate and RCLM to elucidate the causal pathways to fractures. Interventional trials are needed to determine whether a core-focused program targeting VAT (aerobic VAT reduction + core/balance training, with adequate protein/EAA nutrition) reduces falls and secondary fractures more effectively than a limb-focused strategy alone. Drug stratification analyses (e.g., GLP-1 receptor antagonists, SGLT2 inhibitors, insulin) and component-based treatment pathways should be used to assess the impact on A/G, TL, VAT estimate, RCLM, and clinical outcomes. Finally, predictive modeling integrating A/G ratio, TL fat ratio, VAT estimate, and RCLM (including external validation and comparison with FRAX-like tools) may improve risk stratification in patients with type 2 diabetes.

## 5. Conclusions

In this cohort of older adults with hip fracture, T2DM was associated with greater central adiposity, whereas ALMI did not significantly differ between groups. Given the modest sample size, small differences in lean mass distribution cannot be excluded. These findings suggest that fat distribution patterns may represent an important compositional feature in T2DM-related fracture populations. However, prospective studies incorporating functional measures and bone-quality assessments are required to confirm these observations and clarify their clinical implications.

## Figures and Tables

**Table 1:** Baseline characteristics by diabetes status.

T2DM Group *n* = 40; Non-DM Group *n* = 59				
Variable	T2DM Mean ± SD	Non-DM Mean ± SD	*p*-Value	SMD
**Demographics**				
Age (years)	79.62 ± 8.82	78.69 ± 9.86	0.625	0.10
Height (m)	1.56 ± 0.05	1.54 ± 0.06	0.119	0.31
Weight (kg)	56.09 ± 10.13	51.52 ± 10.85	0.036	0.43
BMI (kg/m^2^)	23.14 ± 3.84	21.80 ± 4.36	0.110	0.32
HbA1c (%)	7.05 ± 1.27	5.68 ± 0.44	<0.001	1.56
**Body Composition**				
Android/Gynoid fat ratio	1.13 ± 0.15	1.05 ± 0.17	0.030	0.44
Trunk/Limb fat ratio	1.31 ± 0.22	1.19 ± 0.23	0.007	0.56
Estimated VAT (CoreScan estimate, cm^3^)	658.84 ± 308.81	555.37 ± 259.79	0.086	0.32
Lean mass index (kg/m^2^)	13.28 ± 1.81	12.95 ± 1.63	0.367	−0.41
Appendicular ALMI (kg/m^2^)	5.11 ± 0.94	5.07 ± 0.85	0.821	0.05
Relative central lean mass (%)	61.63 ± 2.82	60.92 ± 3.21	0.254	−0.43

Data are presented as mean ± SD. *p*-values from Welch’s *t*-test. BMI, body mass index; VAT, visceral adipose tissue; ALMI, appendicular lean mass index; SMD, standardized mean difference.

## Data Availability

The data supporting the findings of this study are available from the corresponding author upon reasonable request. The data are not publicly available due to institutional and privacy restrictions.

## Supplementary Materials

The following supporting information can be downloaded at: https://www.mdpi.com/article/10.3390/jcm15062284/s1. Figure S1: VAT estimate vs. android-to-gynoid (A/G) ratio (T2DM subgroup). Figure S2: VAT estimate vs. fat mass index (FMI) (T2DM subgroup). Figure S3: Android-to-gynoid ratio vs. trunk-to-limb fat ratio (T2DM subgroup). Figure S4: FMI vs. total body fat percentage (T2DM subgroup). Figure S5: Appendicular lean mass index (ALMI) vs. lean mass index (LMI) (T2DM subgroup). Supplementary Table S1: Pearson correlation matrix with corresponding *p*-values. Supplementary Table S2: Post hoc achieved-power analysis for primary between-group comparisons.
