# Supplementary material for: Visceral Adiposity, Rather than Reduced Appendicular Lean Mass, Characterizes Elderly Hip Fracture Patients with Type 2 Diabetes: A Cross-Sectional DXA Analysis
Source: J Clin Med. 2026 Mar 17;15(6):2284. doi: 10.3390/jcm15062284 (PMC13026938; doi:10.3390/jcm15062284)
Supplement: Supplementary file 1 [file jcm-15-02284-s001.zip › Figure S1 VAT estimate vs Androidgynoid ratio (T2DM).pdf]

Figure S1. VAT estimate vs Android/gynoid ratio (T2DM)

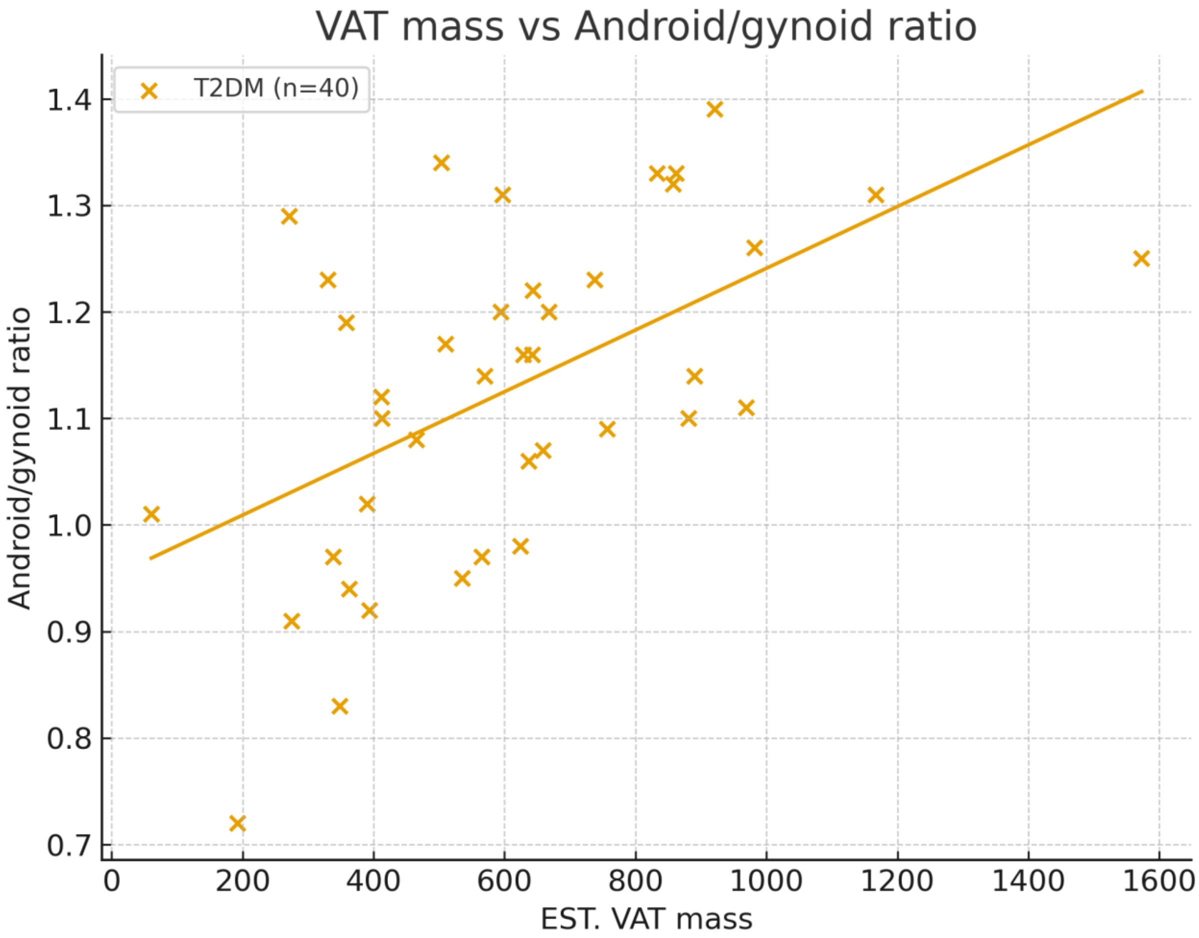

Scatterplot of EST. VAT mass (x-axis) versus Android/gynoid ratio (y-axis) in the T2DM cohort. Pearson's r=0.542, p=0.00031 (n=40).
