# Supplementary material for: Visceral Adiposity, Rather than Reduced Appendicular Lean Mass, Characterizes Elderly Hip Fracture Patients with Type 2 Diabetes: A Cross-Sectional DXA Analysis
Source: J Clin Med. 2026 Mar 17;15(6):2284. doi: 10.3390/jcm15062284 (PMC13026938; doi:10.3390/jcm15062284)
Supplement: Supplementary file 1 [file jcm-15-02284-s001.zip › Figure S3. Android gynoid ratio vs Trunk Limb fat mass ratio (T2DM).pdf]

Figure S3. Android/gynoid ratio vs Trunk/Limb fat mass ratio (T2DM)

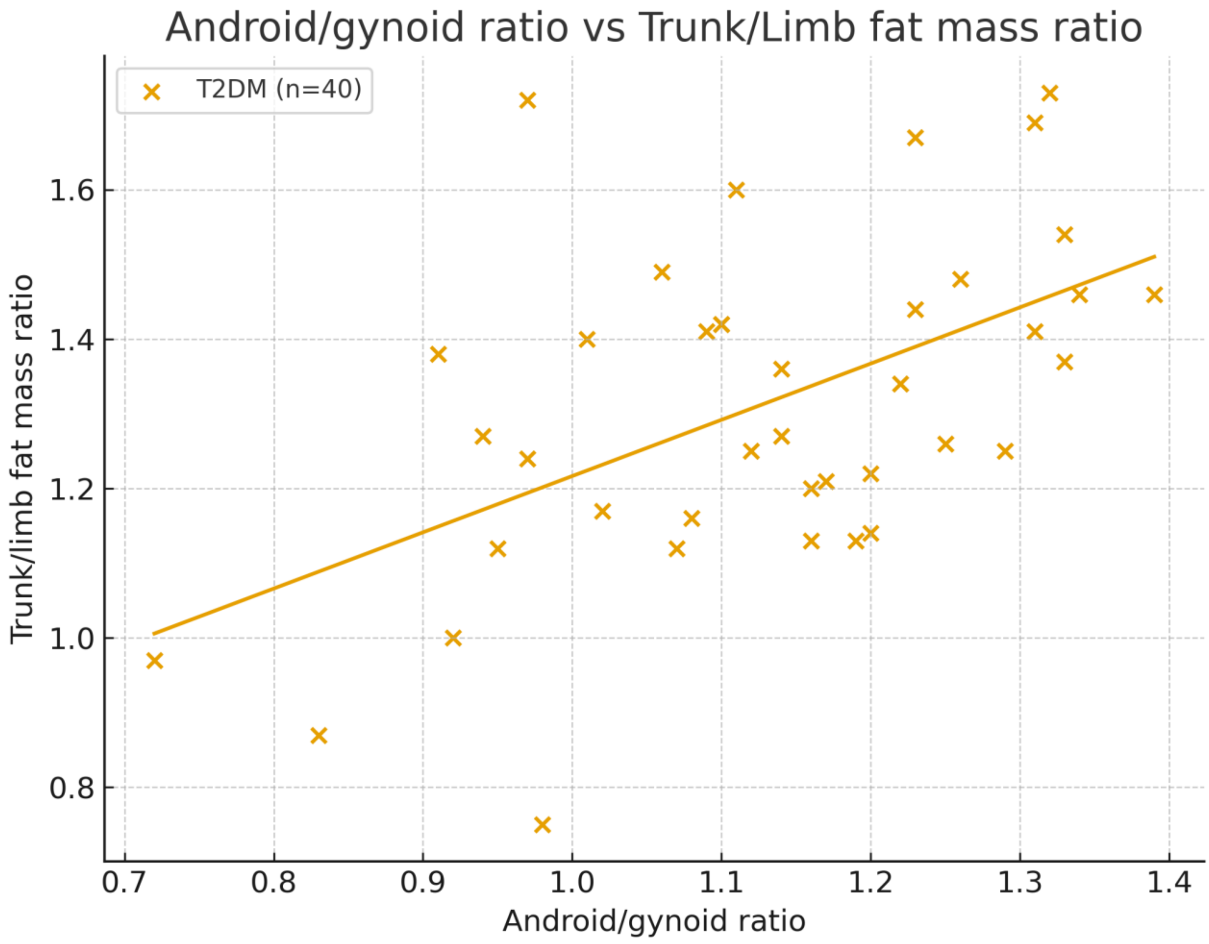

Scatterplot of Android/gynoid ratio versus trunk-to-limb fat mass ratio (unitless) in the T2DM cohort. Pearson's r=0.524, p=0.00052 (n=40).
