# Supplementary material for: Visceral Adiposity, Rather than Reduced Appendicular Lean Mass, Characterizes Elderly Hip Fracture Patients with Type 2 Diabetes: A Cross-Sectional DXA Analysis
Source: J Clin Med. 2026 Mar 17;15(6):2284. doi: 10.3390/jcm15062284 (PMC13026938; doi:10.3390/jcm15062284)
Supplement: Supplementary file 1 [file jcm-15-02284-s001.zip › Supplementary Table S1. DM only correlations(horizontal direction).pdf]

Table S1. Pearson correlation matrix with corresponding p-values

Variable	HbA1c	EST. VAT mass	EST. VAT volume	EST. VAT Area	Android/gynoid ratio	Trunk/limb fat mass ratio	Total body% fat	FMI(kg/M2)	LMI(kg/m2)	appen. LMI	Central lean index (kg/m²)	Relative central lean mass (%)	BMD(F)	BMD(T)
HbA1c	1.000 (p=0.0000)	-0.025 (p=0.8802)	-0.022 (p=0.8936)	-0.022 (p=0.8943)	-0.148 (p=0.3607)	0.054 (p=0.7405)	0.058 (p=0.7224)	-0.086 (p=0.5979)	-0.230 (p=0.1540)	-0.209 (p=0.1965)	0.036 (p=0.8275)	0.168 (p=0.3012)	0.094 (p=0.5705)	-0.004 (p=0.9781)
EST. VAT mass	-0.025 (p=0.8802)	1.000 (p=0.0000)	1.000 (p=0.0000)	1.000 (p=0.0000)	0.542 (p=0.0003)	0.378 (p=0.0161)	0.688 (p=0.0000)	0.788 (p=0.0000)	0.305 (p=0.0561)	0.230 (p=0.1535)	0.010 (p=0.9516)	-0.157 (p=0.3321)	0.160 (p=0.3305)	-0.189 (p=0.2417)
EST. VAT volume	-0.022 (p=0.8936)	1.000 (p=0.0000)	1.000 (p=0.0000)	1.000 (p=0.0000)	0.545 (p=0.0003)	0.383 (p=0.0147)	0.690 (p=0.0000)	0.787 (p=0.0000)	0.306 (p=0.0550)	0.225 (p=0.1620)	0.017 (p=0.9189)	-0.152 (p=0.3501)	0.161 (p=0.3264)	-0.190 (p=0.2414)
EST. VAT Area	-0.022 (p=0.8943)	1.000 (p=0.0000)	1.000 (p=0.0000)	1.000 (p=0.0000)	0.545 (p=0.0003)	0.383 (p=0.0147)	0.689 (p=0.0000)	0.786 (p=0.0000)	0.306 (p=0.0549)	0.226 (p=0.1614)	0.016 (p=0.9202)	-0.152 (p=0.3492)	0.161 (p=0.3260)	-0.189 (p=0.2426)
Android/gynoid ratio	-0.148 (p=0.3607)	0.542 (p=0.0003)	0.545 (p=0.0003)	0.545 (p=0.0003)	1.000 (p=0.0000)	0.524 (p=0.0005)	0.511 (p=0.0008)	0.388 (p=0.0134)	-0.137 (p=0.3994)	-0.096 (p=0.5560)	-0.014 (p=0.9336)	0.061 (p=0.7065)	0.071 (p=0.6683)	-0.370 (p=0.0188)
Trunk/limb fat mass ratio	0.054 (p=0.7405)	0.378 (p=0.0161)	0.383 (p=0.0147)	0.383 (p=0.0147)	0.524 (p=0.0005)	1.000 (p=0.0000)	0.132 (p=0.4173)	-0.043 (p=0.7900)	-0.208 (p=0.1969)	-0.445 (p=0.0040)	0.345 (p=0.0295)	0.450 (p=0.0036)	-0.166 (p=0.3133)	-0.377 (p=0.0166)
Total body% fat	0.058 (p=0.7224)	0.688 (p=0.0000)	0.690 (p=0.0000)	0.689 (p=0.0000)	0.511 (p=0.0008)	0.132 (p=0.4173)	1.000 (p=0.0000)	0.853 (p=0.0000)	0.013 (p=0.9386)	0.012 (p=0.9429)	-0.002 (p=0.9889)	-0.011 (p=0.9456)	0.206 (p=0.2089)	-0.213 (p=0.1878)
FMI(kg/M2)	-0.086 (p=0.5979)	0.788 (p=0.0000)	0.787 (p=0.0000)	0.786 (p=0.0000)	0.388 (p=0.0134)	-0.043 (p=0.7900)	0.853 (p=0.0000)	1.000 (p=0.0000)	0.354 (p=0.0251)	0.448 (p=0.0037)	-0.210 (p=0.1936)	-0.398 (p=0.0110)	0.271 (p=0.0958)	-0.015 (p=0.9268)
LMI(kg/m2)	-0.230 (p=0.1540)	0.305 (p=0.0561)	0.306 (p=0.0550)	0.306 (p=0.0549)	-0.137 (p=0.3994)	-0.208 (p=0.1969)	0.013 (p=0.9386)	0.354 (p=0.0251)	1.000 (p=0.0000)	0.602 (p=0.0000)	0.219 (p=0.1740)	-0.352 (p=0.0258)	0.129 (p=0.4339)	0.212 (p=0.1890)
appen. LMI	-0.209 (p=0.1965)	0.230 (p=0.1535)	0.225 (p=0.1620)	0.226 (p=0.1614)	-0.096 (p=0.5560)	-0.445 (p=0.0040)	0.012 (p=0.9429)	0.448 (p=0.0037)	0.602 (p=0.0000)	1.000 (p=0.0000)	-0.647 (p=0.0000)	-0.959 (p=0.0000)	0.070 (p=0.6701)	0.298 (p=0.0622)
Central lean index (kg/m²)	0.036 (p=0.8275)	0.010 (p=0.9516)	0.017 (p=0.9189)	0.016 (p=0.9202)	-0.014 (p=0.9336)	0.345 (p=0.0295)	-0.002 (p=0.9889)	-0.210 (p=0.1936)	0.219 (p=0.1740)	-0.647 (p=0.0000)	1.000 (p=0.0000)	0.835 (p=0.0000)	0.037 (p=0.8233)	-0.161 (p=0.3207)
Relative central lean mass (%)	0.168 (p=0.3012)	-0.157 (p=0.3321)	-0.152 (p=0.3501)	-0.152 (p=0.3492)	0.061 (p=0.7065)	0.450 (p=0.0036)	-0.011 (p=0.9456)	-0.398 (p=0.0110)	-0.352 (p=0.0258)	-0.959 (p=0.0000)	0.835 (p=0.0000)	1.000 (p=0.0000)	-0.030 (p=0.8565)	-0.260 (p=0.1045)
BMD(F)	0.094 (p=0.5705)	0.160 (p=0.3305)	0.161 (p=0.3264)	0.161 (p=0.3260)	0.071 (p=0.6683)	-0.166 (p=0.3133)	0.206 (p=0.2089)	0.271 (p=0.0958)	0.129 (p=0.4339)	0.070 (p=0.6701)	0.037 (p=0.8233)	-0.030 (p=0.8565)	1.000 (p=0.0000)	0.454 (p=0.0037)
BMD(T)	-0.004 (p=0.9781)	-0.189 (p=0.2417)	-0.190 (p=0.2414)	-0.189 (p=0.2426)	-0.370 (p=0.0188)	-0.377 (p=0.0166)	-0.213 (p=0.1878)	-0.015 (p=0.9268)	0.212 (p=0.1890)	0.298 (p=0.0622)	-0.161 (p=0.3207)	-0.260 (p=0.1045)	0.454 (p=0.0037)	1.000 (p=0.0000)

Abbreviations: VAT, visceral adipose tissue; FMI, fat mass index; ALMI, appendicular lean mass index; CLI, central lean index; RCLM, relative central lean mass; BMD, bone mineral density.
