# Supplementary material for: Visceral Adiposity, Rather than Reduced Appendicular Lean Mass, Characterizes Elderly Hip Fracture Patients with Type 2 Diabetes: A Cross-Sectional DXA Analysis
Source: J Clin Med. 2026 Mar 17;15(6):2284. doi: 10.3390/jcm15062284 (PMC13026938; doi:10.3390/jcm15062284)
Supplement: Supplementary file 1 [file jcm-15-02284-s001.zip › Supplementary Table S2. post-hoc power analysis.pdf]

Supplementary Table S2. Post-hoc power analysis for primary between-group comparisons (α=0.05, two-sided).

**Variable (Table 1)**	**T2DM (n=40) Mean±SD**	**non-DM (n=59) Mean±SD**	**Effect size (Cohen's d)**	**Hedges' g**	**Achieved power (α=0.05, two-sided)**
LMI (kg/m^2)	13.28±1.81	12.95±1.63	0.194	0.192	0.155
RCLM (%)	61.63±2.82	60.92±3.21	0.232	0.230	0.202
A/G ratio	1.13±0.15	1.05±0.17	0.493	0.489	0.664
Trunk/Limb fat ratio	1.31±0.22	1.19±0.23	0.531	0.527	0.728
ALMI (kg/m^2)	5.11±0.94	5.07±0.85	0.045	0.045	0.055
VAT volume (cm^3)	658.84±308.81	555.37±259.79	0.369	0.366	0.430
